# Extrinsic Tooth Enamel Color Changes and Their Relationship with the Quality of Water Consumed 

**DOI:** 10.3390/ijerph9103530

**Published:** 2012-10-05

**Authors:** Kathleen Rebelo de Sousa, Marília Jesus Batista, Juliana Rocha Gonçalves, Maria da Luz Rosário de Sousa

**Affiliations:** 1 Department of Community Dentistry, University of Amazonas, Rua Maceió, 618, apto 902, Edifício Saint Remy, Adrianópolis, CEP 69053-730, Manaus/AM, Brazil; Email: kathleenrebelo@gmail.com; 2 Graduate Program in Dentistry, Piracicaba Dental School, University of Campinas, P.O. Box 52, CEP 13414-903, Piracicaba, SP, Brazil; Email: mariliajbatista@yahoo.com.br (M.J.B.); julianarocha11@uol.com.br (J.R.G.); 3 Department of Community Dentistry, Piracicaba Dental School, University of Campinas, P.O. Box 52, CEP 13414-903, Piracicaba, SP, Brazil

**Keywords:** dental enamel, oral health, water supply

## Abstract

The quality of the consumed drinking water may affect oral health. For example, the presence of iron in drinking water can cause aesthetic problems related to changes in dental enamel color. This study assessed the prevalence of extrinsic enamel color changes and their relationship with the quality of the water in the town of Caapiranga/AM-Brazil. Three hundred and forty six residents of the urban area were examined, and they also answered a questionnaire on eating habits and self-perceived oral health. As the initial results indicated an insufficient number of observations for the application of variance analysis (one-way ANOVA), the Student t test was chosen to compare levels of iron content in the water coming from two sources. The change in tooth color had a prevalence of 5.78% (20 people). The majority of the population (n = 261, 75.43%) consumed well water. Those who presented extrinsic stains were uncomfortable with the appearance of their teeth (15.09%). We conclude that while there is excess of iron in the water in this region of Brazil, no association between extrinsic stains on the enamel and the level of iron in the water was found. There was a low prevalence of extrinsic stains in Caaparinga, being found only in children and adolescents. In the present study, an association between the presence of stains and the consumption of açai was determined, and those who presented them felt uncomfortable about their aesthetics.

## 1. Introduction

Until the end of the last century, water was considered an abundant and practically inexhaustible resource [1]. Brazil was once considered a privileged country in terms of water resources as it has one of the largest reserves of fresh water available in the World. Today, this has been changing since the availability of potable water is decreasing [1], and therefore it is extremely important to study the quality of the drinking water consumed and its possible effects on health, including oral health.

Human actions, the environment and the chemical quality of the water are closely associated [2]. In addition, the water may contain organisms, substances, chemical compounds and elements hazardous to health [3]. The environment and the water may be contaminated with chemicals, causing damage to the biota. These may be metals, semi-metals or non-metals like aluminum, antimony, arsenic, cadmium, lead, copper, cobalt, chromium, iron, manganese, mercury, molybdenum, nickel, selenium and zinc [4]. Iron and manganese ions, commonly found in groundwater, are the most abundant materials in the Earth’s crust [5]. The use of products containing high amounts of iron or iodine may be associated with a substantial black pigmentation of the teeth. Exposure to sulfide, silver nitrate or manganese can cause stains ranging from gray to yellow, brown or black; copper or nickel can produce green stains; cadmium may be associated with pigmentations ranging from yellow to golden-brown [6]. 

The presence of iron in drinking water can cause aesthetic problems related to changes in the color of the dental enamel and buccal mucosa [5]. In Brazil, there are many regions with excess iron in the groundwater [7], but the appearance of extrinsic stains is mainly due to food waste, medicinal substances and bacteria, which form deposits that adhere to the tooth enamel surface [8]. 

Not many studies were found on the association between the presence of iron in the water and changes in tooth color, which makes it of fundamental importance and great value to develop studies on this association, as the stains may affect the aesthetics of these people. 

In 2008, excess iron was found in the population’s drinking water in the district of Caapiranga in the State of Amazonas, as well as the appearance of extrinsic stains, after the collection of water from two sources [9]. Thus, the aim of this study was to assess the prevalence of extrinsic stains and their relation with the concentration of iron in the water, with self-perception concerning tooth aesthetics and with food consumption in the town of Caapiranga-AM. 

## 2. Methodology

This was a cross-sectional study. The town of Caapiranga-AM has a total population of 3,561 according to data from the Caapiranga Municipal Secretary of Health. The distance from Caapiranga to the State capital, Manaus, is 147 km in a straight line, but the only access to the municipality is by water transportation and the distance is about 438 km. Caapiranga has a land area of 9,617 km^2^, which is occupied by the Town Hall where the agencies and public institutions are located, such as executive, legislative and judicial branches, and the various communities that are subordinate to the town’s political administrative council [10].

In Caapiranga, the population is served by a public water supply network offered by COSAMA (Sanitary Company of Amazonas). Nowadays, most residents use wells for water consumption and other activities, and others use water directly from the river. It is noteworthy that there is no water treatment plant (WTP) in the district of Caapiranga-AM. The town is divided into 13 micro-areas, which are assisted by the Family Health Strategy.

Sample size was calculated using the formula in which the prevalence of the phenomenon (color of tooth enamel) would be the most possible (50%), totaling 400 people. These people were divided into 13 micro-areas (subdivision of districts in the town for the Family Health Strategy). All micro-areas were included in the study. We carried out a weighted lottery of the households taking into account the number of households by micro-area, and then all the people in the residences were evaluated. Considering that an average four people lived in each household, 100 households were randomly selected. Data for the sample selection such as number of families and residents were obtained through the Family Health Units (Table 1).

People not resident in the urban area of Caapiranga-AM and people who had changes in tooth color from intrinsic factors such as imperfect amelogenesis, imperfect dentinogenesis, dental fluorosis, erythropoietic porphyria, hyperbilirubinemia, decomposition of blood red cells, supplements containing iron, iodine, potassium, calcium and so on were excluded from the study. Edentulous people were excluded because the study evaluated the presence of extrinsic stains in dentate individuals. 

Enrolled in the study were randomly selected people who agreed to take part by signing a WIC (consent form). A questionnaire was used to obtain data on food consumption, adapted to the local reality, which includes issues of self-perception in relation to oral health. For this study, the question of self-perception in relation to the aesthetics of the teeth was used. The examiner was trained by a reference examiner during a total of 8 hours of theoretical and practical discussions, until at least 90% concordance was obtained according to standard WHO recommendations [11]. Oral examinations were performed in home visits by an examiner with the help of an oral health assistant. Clinical examination was performed with the aid of a disposable dental mirror under natural light, with the dentist properly protected with complete Personal Protective Equipment (PPE). The people who presented changes in tooth color were informed about the problem and referred to the “Selestino André de Souza” Basic Health Unit for a dental consultation.

Water samples were collected in resistant plastic vials, which were chemically inert and had a tight seal. The samples were collected from 8th to 15th August in 2008. A collection was performed for each water supply in each family residence selected, and if a residence had more than one type of water supply, the corresponding samples were collected. Each sample had a minimum volume of water (300 mL) to make the analysis possible and thereby assess the presence of iron metal. The collection, security and measurement equipment was selected for the purpose of the sampling required. The internal parts of the bottles and covers should not be touched with the hands. The sample bottles were opened only during the time required to fill them, and were kept protected from sunlight. Once collected, the water samples were transported to the laboratory so that their integrity could be guaranteed and their preservation time respected [12]. A chemical analysis laboratory in Manaus, AM was used to perform the water analyses using the Atomic Absorption Spectrometry method to determine the concentration of iron. The appropriate references followed were: Standard Methods-21st Edition, 1060 B. Collection of Samples and 1060 C. Sample Storage and Preservation.

Chi-square and Fisher’s exact tests were performed to verify the association between the iron content in the water and extrinsic stains. As the first results indicated an insufficient number of observations for the application of analysis of variance (one-way ANOVA), instead the Student t test was chosen to compare levels of iron content from two water sources. The data were tabulated in Excel and converted to SPSS 17.0 for analyzes. To analyze the content of iron in the water and the presence of stains on the teeth, the variable iron content was divided at normal iron content (up to 0.30 mg/L) and iron content above normal (>0.30 mg/L). The answers to the question of self-perception concerning aesthetic were divided into good and excellent (their appearance does not bother them) and fair, poor and very poor self-perception (their appearance bothers them). This study had its project was submitted to and approved by the National Council of Research Ethics (CEP ESA/UEA 068-08).

## 3. Results

Three hundred and forty six (346) residents of Caapiranga were examined (a loss of 13.5% of the sample) in the 13 micro-areas of the town (Table 1). 

**Table 1:** Distribution of the number of families, residents and people in the study micro-areas (Caapiranga, AM, Brazil, 2011).

Districts		Families (n)	Residents (n)	Sample selected (n)
1	Downtown 1	73	305	32
2	Downtown 2	44	179	20
3	Downtown 3	60	316	36
4	St. Luzia 1	75	401	29
5	St. Raimundo	67	281	32
6	Countryside áreas	56	231	22
7	St. Luzia 2	81	315	15
8	St. Luzia 3	64	300	34
9	St. Antonio 1	48	204	23
10	St Antonio 2	61	278	23
11	St. Atonio 3	47	235	22
12	St Antonio 4	68	342	38
13	Novo Horizonte	40	174	20
TOTAL		784	3,561	346

The losses were because these people were not found at home during the researchers’ visit. As for age, there was a wide variation (1–87 years) with an average of 23.88 years, and 51.4% (n = 178) were female. Twenty individuals presented extrinsic stains (5.78%) and there was no significant difference regarding gender. Extrinsic stains enamel were found only in younger people 4–13 years old (Table 2).

**Table 2:** Number of subjects, mean age and standard deviation according to gender and according to the presence or absence of spots, Caapiranga, AM, Brazil, 2011.

Gender		Spots		General
		No	Yes	
	n (%)	169 (94.94%)	9 (5.06%)	178 (100.00%)
Female	Mean Age (SD)	25.42 (18.43)	8.67 (2.06)	24.57 (18.33)
	n (%)	157 (93.45%)	11 (6.55%)	168 (100.00%)
Male	Mean Age (SD)	24.24 (18.34)	7.73 (2.61)	23.15 (18.20)
Total	n total (%)	326 (94.22%)	20 (5.78%)	346 (100.00%)
	Mean Age (SD)	24.85 (18.37)	8.15 (2.37)	23.88 (18.26)

Note: Chi square test by gender.

In the northern region, average annual temperatures can exceed 25 °C [13] weather leads people to consume a lot of water, as found in this study in which water consumption varied from 1 to 20 times a day, mostly well water (n = 261, 75.43%). The presence of stains (20 individuals = 5.78%) was observed among those who consumed well water (Table 3). 

**Table 3:** Numbers of subjects according toextrinsicenamelstainoccurrence in accordance withthe source of thewater, Caapiranga, AM, Brazil, 2011.

Water procedence	Spots		Total (%)
	No	Yes	
Well	241	20	261 (75.43%)
Well and spring water	6	0	6 (1.73%)
Well and tap water	44	0	44 (12.72%)
Well and river water	9	0	9 (2.61%)
Net	26	0	26 (7.51%)
Total	326	20	346 (100.00%)

According to the chemical analysis of the water, iron was the only component found in excess. For the analysis, 89 samples were used. It was decided that the samples from secondary sources (second source of water used for drinking), which were few in number (n = 13), should be disregarded for the analysis and discussion. In addition, 11 samples were lost because the residents were absent at the time of collection (Table 4).

**Table 4:** Number of samples, average and standard deviation of iron content in water source, Caapiranga, AM, Brazil, 2011.

Water	Iron content (mg/L)		
	N	Average	Standard Deviation
Well	78	0.251	0.40996
Tap water	8	0.839	0.91180
River	1	0.19 (1)	(2)
Total	87	0.304597701	0.497416125

On the recommendation of Decree 518 [13], the limit of iron allowed for water consumption is 0.30 mg/L and the levels found in the town ranged from 0.01 to 2.45. Thus, a test was carried out to relate the iron content and the presence or absence of stains, according to Table 5.

**Table 5:** Number (%) of subjects with and without spots in accordance of iron content in water, Caapiranga, AM, Brazil, 2011.

Iron	Spots		
	No (n = 326)	Yes (n = 20)	Total
Up to 0.3	239 (93.73%)	16 (6.27%)	255 (100.00%)
More than 0.3	87 (95.60%)	4 (4.40%)	91 (100.00%)

Among other possible causes for the stains, the consumption of juice, coffee, tea and açai was analyzed (Table 6). To evaluate the association between the occurrence of stains and the consumption of juice, tea, coffee and açai, the Fisher’s exact test was used.

**Table 6:** Number and percentage of subjects according to presence or absence of extrinsic enamel stains according to the consumption or not juice, coffee, tea, and açai, Caapiranga, AM, Brazil, 2011.

Drink	Consumption	Spots		
		No	Yes	Total
Juice	No	72 (94.74%)	4 (5.26%)	76 (100.00%)
	Yes	254 (94.07%)	16 (5.93%)	270 (100.00%)
Total		326	20	346
Tea	No	283 (94.65%)	16 (5.35%)	299 (100.00%)
	Yes	43 (91.49%)	4 (8.51%)	47 (100.00%)
Total		326	20	346
Coffee	No	67 (98.53%)	1 (1.47%)	68 (100.00%)
	Yes	259 (93.17%)	19 (6.83%)	278 (100.00%)
Total		326	20	346
Açai	No	262 (96.32%)	**10 (3.68%) a**	272 (100.00%)
	Yes	64 (86.49%)	**10 (13.51%) b**	74 (100.00%)
Total		326	20	346

Note: different letters in line show statistical difference (*p *< 0.05).

**Table 7:** Numbers of subjects and perceptions of teeth appearance (with and without spots), Caapiranga, AM, Brazil, 2011.

Spots	Teeth appereance		Total
	Not disturbed	Disturbed	
No	236 (98.33%)	90 (84.91%)	326
Yes	4 (1.67%) a	16 (15.09%) b	20
Total	240 (100.00%)	106 (100.00%)	346

Note: different letters in line show statistical difference (*p *≤ 0.05).

The percentage of stains among those who consumed açai was significantly higher than the percentage of spots observed among those who did not. The data regarding the appearance of the teeth obtained by the self-perception exam is given in Table 7 above. In this case, those who presented stains were uncomfortable with the appearance of their teeth.

## 4. Discussion

Several people who sought care at the Caapiranga, AM, health units inquired about the frequent appearance of tooth stains, claiming that these stains undermined their aesthetics, and asking for their removal. This new study obtained real data on the quality of water at the source of the water consumed at each residence, bearing in mind that there was no association between tooth staining, type of water consumed and the presence of iron in the water. However, in addition to verifying that people who had extrinsic stains were uncomfortable with the aesthetics of their teeth, there was an association between the stains and the consumption of açai. 

In relation to the recommendations of Decree 518 of 2004 [14], excess iron was found in 10 of the 13 micro-areas, which points to the importance of investigating the quality of the drinking water consumed by the population of Caapiranga, since the presence of excess iron in the water causes change in the color and an unpleasant taste [3]. 

Dental stains can be produced by several factors, especially when they are associated with one another. A study observed that lactoferrin (a bactericidal protein found in saliva) and the iron present in human saliva produce an increase in staining of the tooth enamel [15]. Another factor that may be associated with tooth staining is high consumption of tea, coffee and wine [16]. According to some authors [17], these beverages contain a considerable amount of tannic acid, which can produce staining. In the present study, no association was observed between the iron content found in the water used by the town residents and the presence of dental stains, unlike what was reported by researchers in 2004 [18], who found an association between iron in the water sources and tooth staining in the residents of Nepal. However, the presence of extrinsic stains can be associated to dietary habits [19]. The consumption of açai berry is a common eating habit in the North and, according to Menezes, Torres and Srur [20], it contains iron (4.5 mg/100 g of pulp). In this study, the percentage of stains among those who consume açai was significantly higher than the percentage observed among those who do not, and there was no relationship between the consumption of tea, juice and coffee and the appearance of extrinsic stains. The presence of extrinsic stains in this study was significant for age as it was found only in young people 4–13 years old. No studies were found that would explain the presence of extrinsic stains only in young people.

Most of the people examined consumed well water (n = 261), but there was no significant difference between the presence of stains and the type of water consumed. Excess iron can cause changes in the water color and taste, and it can also cause stains on clothing and sanitary utensils, so it may be assumed that the reason why little water from the Public Supply Network is consumed by most of the population, as in some homes they have at least two types of water source available (the well and the public supply network).

It must be considered that in the drinking water distribution systems, the water quality may suffer a series of changes from the moment it leaves the treatment plant to the water at the user’s tap. Such changes can be caused by chemical and biological variations or losses of system integrity [21]. These changes in the quality of the water that comes to the user’s tap may be one explanation for the contamination found in the Public Supply Network water. During the time between when the water leaves the source up to the final consumer, there may be a change in its quality due to the aggregation of contaminants, poor sanitation and poor water quality. The consumption of poor quality water can cause serious health problems such as waterborne diarrheal diseases, such as typhoid, cholera, salmonellosis, shigellosis and other gastroenteritis, polio, hepatitis A, worms, amoebiasis and giardiasis. In developing countries, it has been responsible for several outbreaks and high infant mortality rates [21]. It is therefore necessary to control the quality of the drinking water distributed to the population of Caapiranga.

In Caapiranga, excess iron was found in the public water supply, but for others [22] low concentrations of metals are often detected in water. However, this fact is misleading because, as a result of the anaerobic conditions, there is formation of insoluble salts and removal by sedimentation. Metals in the water distribution system may result from the variability of the quality of the water in the distribution system or be related to it [23]. We emphasize that seasonal period (excessive rain and drought) was not taken in consideration. 

For this reason, water treatment is recommended as a guarantee to health for the elimination of possible contaminants as well as the filtration and disinfection that reduce the possibility of transmission of pathogens. In addition to the control of the water of the municipal supply network, it is fundamental to raise people’s awareness on the importance of maintaining the wells, as most of the population in this northern municipality uses water wells for consumption and several uses of water. The wells play a key role in the water supply for low-income families, including families in the interior of the state of Amazonas, and they should follow technical and operational standards to prevent contamination that could compromise the quality of the groundwater, thus posing a risk to people’s health [24]. This did not occur in the town that was the aim of this study, since some wells showed the presence of iron above the standards set by Ministry of Health [14].

Individuals with stains, who were between 4 and 13 years old, were uncomfortable with the perception of the aesthetics of their teeth and, according to other researchers [25], for adolescents, oral health is associated with good looks. The use of subjective indicators related to the perception about oral health in adolescents can contribute with the evaluation and health education since they favor more careful planning and directed at this population [26]. Knowing the negative perception about aesthetics in children and adolescents in Caaparinga, it is possible to work on the issue of prevention of dental staining as a need perceived by them, and then expand to other topics of oral health promotion.

This study was intended to be exploratory for further research about the possible effects of excess iron in the water, as well as the cause of the presence of extrinsic stains, since the iron in this study was not associated with the presence of stains. However, we highlight that the prevalence was low and probably we did not find an association due to sample size. 

## 5. Conclusions

There was low prevalence of extrinsic stains, being found only in children and adolescents and no association was found between the extrinsic stains and the level of iron in the water in Caaparinga. In the present study, there was a positive association between the consumption of acai and the presence of stains and those who presented them felt more uncomfortable about their aesthetics. 
